# Investigating an Integrated Sensor Fusion System for Mental Fatigue Assessment for Demanding Maritime Operations

**DOI:** 10.3390/s20092588

**Published:** 2020-05-02

**Authors:** Thiago Gabriel Monteiro, Guoyuan Li, Charlotte Skourup, Houxiang Zhang

**Affiliations:** 1 Department of Ocean Operations and Civil Engineering, Norwegian University of Science and Technology, 6009 Ålesund, Norway; thiago.g.monteiro@ntnu.no (T.G.M.); guoyuan.li@ntnu.no (G.L.); 2 Products and Services R&D, Oil, Gas and Chemicals, ABB AS, 0666 Oslo, Norway; charlotte.skourup@no.abb.com

**Keywords:** physiological sensors, mental fatigue, maritime operations, deep learning

## Abstract

Human-related issues are currently the most significant factor in maritime causalities, especially in demanding operations that require coordination between two or more vessels and/or other maritime structures. Some of these human-related issues include incorrect, incomplete, or nonexistent following of procedures; lack of situational awareness; and physical or mental fatigue. Among these, mental fatigue is especially dangerous, due to its capacity to reduce reaction time, interfere in the decision-making process, and affect situational awareness. Mental fatigue is also especially hard to identify and quantify. Self-assessment of mental fatigue may not be reliable and few studies have assessed mental fatigue in maritime operations, especially in real time. In this work we propose an integrated sensor fusion system for mental fatigue assessment using physiological sensors and convolutional neural networks. We show, by using a simulated navigation experiment, how data from different sensors can be fused into a robust mental fatigue assessment tool, capable of achieving up to 100% detection accuracy for single-subject classification. Additionally, the use of different sensors seems to favor the representation of the transition between mental fatigue states.

## 1. Introduction

In recent years, maritime operations have become more demanding every day. Maritime operations now include tasks such as anchor-handling operations at depths of several thousand meters, precise installation of sub-sea modules weighing hundreds of tons, and platform support in icy and cold environments in northern regions. The level of complexity of these operations increases even further when they require coordination among the professionals operating vessels, cranes, winches, and/or remotely operated vehicles. These factors also increase the damage that accidents can cause.

Recent studies pointed out that human and organizational factors are the most significant causes of maritime accidents [1], and the maritime sector generally considers human related factors to be the main factors contributing to accidents [2]. Among the possible causes of human error, the most common ones are related to situational awareness challenges. Endsley classically defines situational awareness as “the perception of the elements in the environment within a volume of space and time, the comprehension of their meaning, and the projection of their status in the near future” [3]. Sneddon et al. studied the relationships between situational awareness and fatigue, sleep disruption, and stress, and how these factors affect maritime operators’ unsafe behaviors, accidents, and near-misses [4]. The research showed that, “Higher levels of stress and fatigue are linked to lower levels of situational awareness, which in turn are indicative of increased participation in unsafe work behaviors, and higher accident risk”. Stress [5] and sleep quality [6] are known factors influencing the performance of human operators. They are also direct causes of fatigue in maritime operators, which in turn is one of the main causes of maritime accidents [7].

The International Maritime Organization defines fatigue as a “reduction in physical and mental capability as the result of physical, mental or emotional exertion which may impair nearly all physical abilities including strength, speed, reaction time, coordination, decision making and balance” [7]. Among common fatigue effects, the literature points to the tendency to accept greater risks while having reduced cognitive capacity to deal with them [8]; to the variability in motivation and work efficiency; and to lapses of vigilance [9]. “Diminished capacity” generally is a core part of the physiological definition of fatigue [10].

The fatigue experienced by maritime operators can have several causes. Researchers once believed that operators alone were responsible for their own fatigue, due to poor lifestyles and personal habits, and that strong will and coffee were enough to overcome fatigue [10]. However, research shows that harsh working conditions characterized by permanent noise, vibration, heat, and bad weather; long periods away from home and family; the difficulty separating leisure and work while on board; and cultural differences in the working environment when people of different cultural backgrounds work together in a stressful environment are all causes of fatigue over which operators have little control [11].

The most influential document regarding fatigue issues in the maritime environment is the “Guidance on Fatigue Mitigation and Management” [7]. It provides information about “the nature of fatigue, its causes, preventive measures and countermeasures” as it relates to vessel safety. While it shines light on the role of fatigue in maritime accidents, the International Maritime Organization guidance does not tackle fatigue assessment in a way that operators or their supervisor can use. It names loss of appetite, sluggish feelings, needless worry, reduced motivation, and attention span as among the signs of fatigue. It acknowledges that people usually have difficulty recognizing fatigue in themselves, not least because fatigue impairs judgment and problem solving capacities. Yet the document provides no alternative to self-diagnosis of fatigue, in spite of the fact that signals of fatigue can be very subjective and hard to spot.

The approaches for assessing fatigue can be divided in two main categories: subjective and objective approaches [12]. The subjective evaluations include methods such the NASA Task Load Index [13], the Karolinska Sleepiness Scale [14], and diaries and surveys [15]. Although these subjective approaches can achieve good results in assessing fatigue state, their self-reported nature and methodological issues about how participants provide data can also make them biased. Regarding the objective assessment of fatigue, there are three main ways that fatigue symptoms can manifest: physiologically, emotionally, and mentally [7]. Physiological symptoms include all body-related signals of fatigue, such as difficulty with hand-eye coordination skills, headaches, rapid breathing, irregular heartbeats, and heart palpitations. Emotional symptoms include all the emotion-related signals of fatigue, such as needless worry, abrupt mood changes, reduced work motivation, and anti-social behavior. Mental symptoms include all the reasoning and thinking-related signals of fatigue, such as reduced attention span, difficulty concentrating and thinking, and slow response to normal, abnormal, and emergency situations.

Among the previously described symptoms of fatigue, the most reliable ones to monitor are the operator’s physiological signals, including respiration, electrooculogram (EOG), electromyogram (EMG), electrocardiogram (ECG), and electroencephalogram (EEG) [16]. Nilsson et al. [17] presented a list of physical symptoms of fatigue, which can be used together with physiological studies to decide the most suitable kind of monitoring equipment for this application. An integrated multiple sensor facility can be used for monitoring different body parts of the operator, from where fatigue can easily manifest. For example, wearable or environmental fixed sensors can be used for detecting breath intake, measuring heart rate, tracking eye movement, and so on. Although individual sensors can provide some understanding of the operators’ working state, they can be insufficient to reliably evaluate operational risks. Additionally, the use of individual sensors can be problematic, since there may be limitations in spatial and temporal coverage, imprecision, and uncertainty [18]. The use of sensor fusion techniques is one of the most suitable ways to handle data from disparate sensors [19] and can be used for developing a cross-modal cognition algorithm which will increase the quality and the usefulness of the sensors’ data, generating more accurate and complete model descriptions.

A small number of studies have investigated the effects of fatigue in vessel simulators [20,21] or on real vessels [22,23]. Even fewer studies have developed methods to assess fatigue while operations are taking place, in spite of the fact that real-time data is the only way to prevent accidents. Most methods of fatigue assessment work a posteriori, through the use of questionnaires. Given the limitations of currently available fatigue assessment methods for preventing maritime accidents, a novel approach needs to be developed. In this work we propose the use of a physiological sensor framework and an artificial neural network to perform mental fatigue assessment during maritime operations. We propose an approach for developing a mental fatigue scale, which can be used as a prognostic tool to reduce accident risks. The underlying algorithm requires little computational effort and can be applied in real time. The proposed approach was preliminarily tested by using as a case study a simulated navigation task carried out in a vessel simulator. The experimental results showed the superior power of EEG for mental fatigue detection over other physiological sensors, but also suggests that combining different sensors can be beneficial for capturing the transitions between mental fatigue states.

## 2. Materials and Methods

### 2.1. Sensor Framework

**Figure 1:**
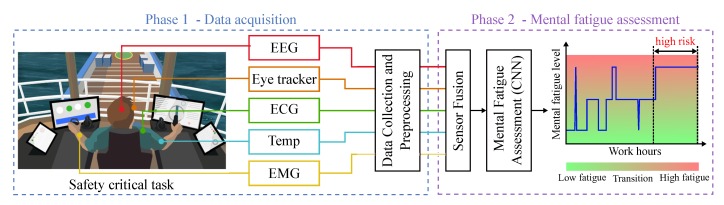
Proposed concept for fatigue assessment.

In this paper we are especially interested in using physiological sensors to measure mental fatigue (MF), due to their capacity to reduce reaction time, interfere in the decision-making process, and affect situational awareness. We propose a sensor framework which can be used to assess the MF state of human operators during demanding maritime operations, including crane operation, remote piloting, dynamic positioning operation, navigation, etc. The proposed concept is shown in Figure 1 and its workflow can be subdivided in two main phases:

- **Phase 1—data acquisition.** During this phase a set of sensors is used to collect physiological data from the operator. The data are collected from disparate sensors and centralized by a micro-controller. During this phase the data are also preprocessed to remove noise and unwanted artifacts that can disturb the fusion and classification processes.
- **Phase 2—mental fatigue assessment.** During this phase a sensor fusion algorithm is applied to the preprocessed data. This algorithm is responsible for fusing the disparate data channels and outputting an MF indicator. This MF indicator is registered and can be used to evaluate the risk level in the operation.

Below we briefly describe the most relevant building blocks of this framework.

### 2.2. Sensors Setup

**Figure 2:**
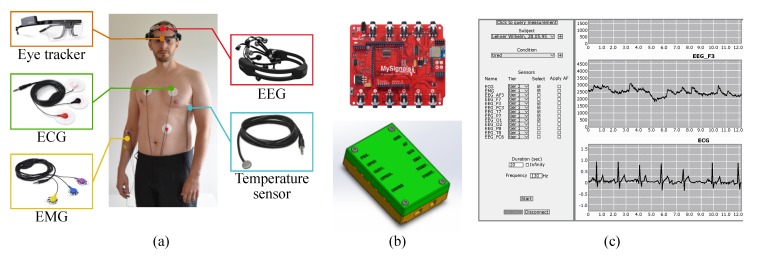
Sensor setup. (**a**) Selected sensors and how to wear them. (**b**) MySignals Arduino shield and protective case. (**c**) Data acquisition application implemented in Java.

Our proposed sensor setup is composed of five sensors: ECG, EMG, body temperature sensor, EEG, and eye tracker. The usage of the sensors is depicted in Figure 2a.

#### 2.2.1. ECG

The ECG is one of the most commonly used sensors in medicine due to its low degree of invasiveness. It also enables accurate diagnosis of diverse cardiac pathologies, such as myocardial ischemia, infarction, and palpitations. Additionally, it provides relevant information about fatigue, since a person’s heart rate varies significantly while in different states of tiredness [24]. We use a MySignals three leads ECG system [25].

#### 2.2.2. EMG

EMGs detect skeletal muscle activity by measuring the difference of potential between two electrodes placed on the muscle, which characterizes the muscular contraction. EMGs are used to diagnose neuromuscular diseases and in applications related to prosthetic control, grasp recognition, and human–computer interaction [26]. We use the EMG to track how muscle contraction intensity varies during the performed operation, which can decrease as a consequence of an increase in an individual fatigue state. Like our ECG, our EMG system has three leads and is made by MySignals.

#### 2.2.3. Body Temperature Sensor

Body temperature sensors have been in use for centuries and remain one of the most accessible physiological sensors available. The body temperature has a close relation to circadian rhythms and can fluctuate as the body activity level changes throughout a period of time. That can be an indication of changes in an individual fatigue state [27]. The body temperature sensor is applied to the operator’s thorax, under the armpit. The sensor is made by MySignals.

#### 2.2.4. EEG

The EEG measures brain electrical activity by tracking voltage fluctuations resulting from ionic current within neurons [28]. EEG is probably the most used physiological measurement of MF due to the clear relation between the power spectrum characteristics in different frequency bands and MF levels [29]. We use the Emotiv EPOC+, an EEG headset with 14 channels made by Emotiv [30].

#### 2.2.5. Eye Tracker

The eye tracker adds a wide range of possible analyses regarding MF and drowsiness. Data regarding eye movement, blinking rate, interval between opening and closing the eyes, and attention span can be used to assess states of MF, drowsiness, or situational awareness [31]. We use an eye tracker Tobii Pro Glasses 2 [32].

#### 2.2.6. Data Centralization

In order to collect and store data from these sensors in a centralized way, an Arduino is used as a micro-controller. The Arduino works as the interface between the sensors and the data handling application running in a laptop. An Arduino shield provided by MySignals acts as an input interface for the ECG, EMG, and body temperature sensor. Since the sensors require wires connecting the user to the shield, we also designed a lightweight and compact protective case for containing the Arduino and the MySignals shield (Figure 2b). This case can be easily carried in a belt or vest. The Arduino can be connected to the laptop using a USB cable via a serial port for bigger bandwidth or via Wi-Fi for better mobility.

The EEG headset from Emotiv and the eye tracker glasses from Tobii have their own specific Wi-Fi communication protocols and can be connected to the laptop directly, without the need to interface directly with the Arduino. The data from sensors is handled by a Java application running in a laptop (Figure 2c).

### 2.3. Data Collection and Preprocessing

When dealing with disparate sensors, different kinds of data can be obtained. It is important to bring these data to a common domain to facilitate the data fusion process. In our MF assessment approach, we handle the data from all sensors in the time domain, as time-series. Bellow we describe which kind of data can be obtained from each sensor and how we bring them to the time domain to perform the data fusion.

The eye tracker provides valuable insights on focus and attention levels of the operator. It records and analyzes information about the eyes and their field of view. It is possible to use information such as pupil diameter and movement and blinking rate to assess concentration and tiredness. Relying on the recorded field of view and the definition of areas of interest (such as control panels, outside environment, and displays), it is possible to define gaze and screening patterns to evaluate focus and situational awareness. In our analysis we are not going to employ gaze-related variables, since they provide little insight into MF levels.

When analyzing EEG data, one very common approach is to decompose the signal from each electrode in the main frequency bands of clinical interest for MF detection; namely, delta (0.3–4 Hz), theta (4–8 Hz), alpha (8–13 Hz), and beta (13–30 Hz) sub bands [33]. This decomposition can be done in the time-frequency domain using discrete wavelet transform, which allows spectral analysis while maintaining time correlation.

The three remaining sensors (ECG, EMG, and temperature) can be analyzed directly as time-series. Changes in the signal patterns of each of these individual sensors may not be enough to characterize with a high level of certainty the progression of an individual’s MF state. The corroboration of information from these disparate sensors, though, can increase the level of confidence in the MF state detection.

### 2.4. Sensor Fusion

The use of individual sensors can be problematic, given their propensity for deprivation, limited spatial and temporal coverage, imprecision, and uncertainty [18]. When sensor fusion techniques are applied to sensory data from different sources, we expect the desired output to be more robust and reliable [34].

Sensor fusion applications can be characterized by several components. The fusion levels can be classified as low or raw data fusion, intermediate or feature level fusion, and high or decision fusion [18]. At the raw data level, data from different sensors can be analyzed in a centralized manner. In order to overcome the discrepancy constraints in terms of mathematical complexity of sensor data, fusion on the feature level can extract features to reduce the communication bandwidth requirements. Estimation and filtering techniques can be used here. Fusion at the decision level increases the reliability of decision making. In this case, a decentralized approach is more appropriate.

Regarding the way sensors interact with each other, sensor fusion applications can be classified as cooperative, competitive, and complementary, and these categories are not mutually exclusive [35]. Sensor fusion application can also be classified regarding the system architecture. A wide range of architectures can be identified on the literature, from the most classical ones, such as JDL fusion architecture [36] to most recent ones, such as multiple functional neural fuzzy networks and deep convolutional neural network (CNN) [37].

**Figure 3:**
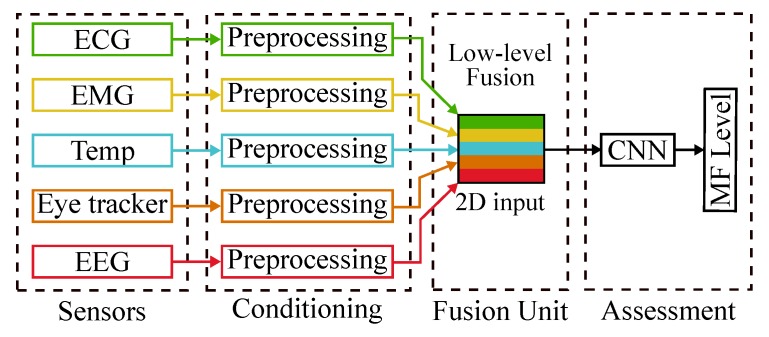
Proposed sensor fusion structure.

In our study, we used different physiological sensors in a cooperative way and performed the data fusion in a centralized manner. We opted to use low-level (or raw data) fusion in the multivariate time-series data obtained from the physiological sensors. The low-level fusion allows the neural network responsible for the MF assessment to select relevant features to describe the different MF states by itself. Figure 3 shows our sensor fusion structure.

**Figure 4:**
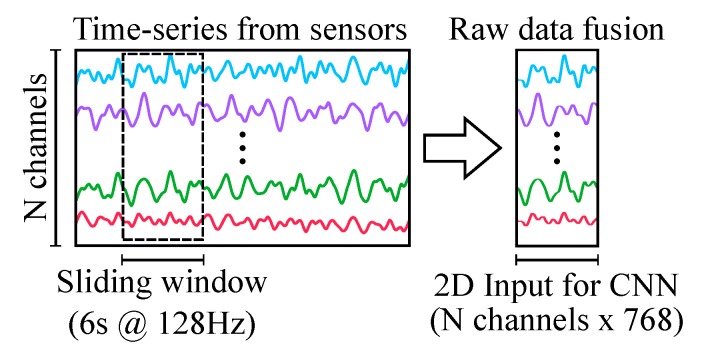
Raw data fusion scheme. The data from different sensors’ channels is segmented using a sliding window and concatenated as a 2D input for the CNN.

The multivariate time-series data obtained from the physiological sensors were sampled at 128 Hz and segmented in 6 s segments with 2 s of overlap between consecutive segments. The segments from different sensors were grouped as an input matrix for the sensor fusion algorithm, as shown in Figure 4.

### 2.5. Mental Fatigue Assessment

Recently, a number of papers have been published regarding the use of physiological sensors and deep learning for assessing MF, drowsiness, and mental workload [38,39,40,41]. Although some of these previous works present solid results, they usually also present a complex neural network structure, containing up to several million parameters, and computationally expensive algorithms. In this work we are more interested in investigating the MF assessment framework as a whole and its possible application in the maritime industry. By envisioning real-time applications, we also wanted to ensure a simple assessment algorithm, which could be trained and applied with minimal computational effort. Considering these aspects, we opted to use a conventional CNN for our MF assessment algorithm. This choice seemed natural, since the fused signal from different sensors can be interpreted as a 2-dimensional array, which is the kind of input the CNN was originally developed to handle. Additionally, the ability to filter the raw signals and extract key features while reducing the dimensions of data makes the CNN a good method to fuse the time-series data from our framework.

Besides one input and one output layer, CNNs have multiple hidden layers responsible for feature extraction and dimension reduction. The hidden layers can be of two types: convolutional layers or pooling layers. Convolutional layers are the ones responsible for the feature extraction task. They do so by convolving learnable kernels across the width and height of the input data to produce an activation map of that filter. The discrete 2-dimensional convolution is defined as

(1) $$S\left(i,j\right)=\left(I\cdot K\right)\left(i,j\right)=\sum_{m}\sum_{n}I\left(m,n\right)K\left(i-m,j-n\right)$$

where *I* is the input data, *K* is a kernel, *i* and *j* are the discretized time indexes, and *m* and *n* are the number of elements in each dimension of the input data. After convolution, the obtained linear filters are processed by a non-linear activation function, before the pooling operation. Common choices for this activation function are sigmoid and ReLU. Pooling layers are responsible for the dimension reduction task. They do so by combining the input of a cluster of nodes in a single output. Common choices for the pooling operation are maximum and average pooling. A CNN can have any number of convolutional and pooling layers.

After the convolutional and pooling layers, a fully connected layer is used to converge the obtained feature maps in a flat input to an output layer. The output layer uses as softmax function to classify the input sensor data in one of the desired classes. The unit softmax function is defined as

(2) $$Softmax\left(x_{i}\right)=\frac{exp(x_{i})}{\sum_{j}^{K}exp\left(x_{j}\right)},\;for\;i=1,\ldots ,K$$

**Figure 5:**
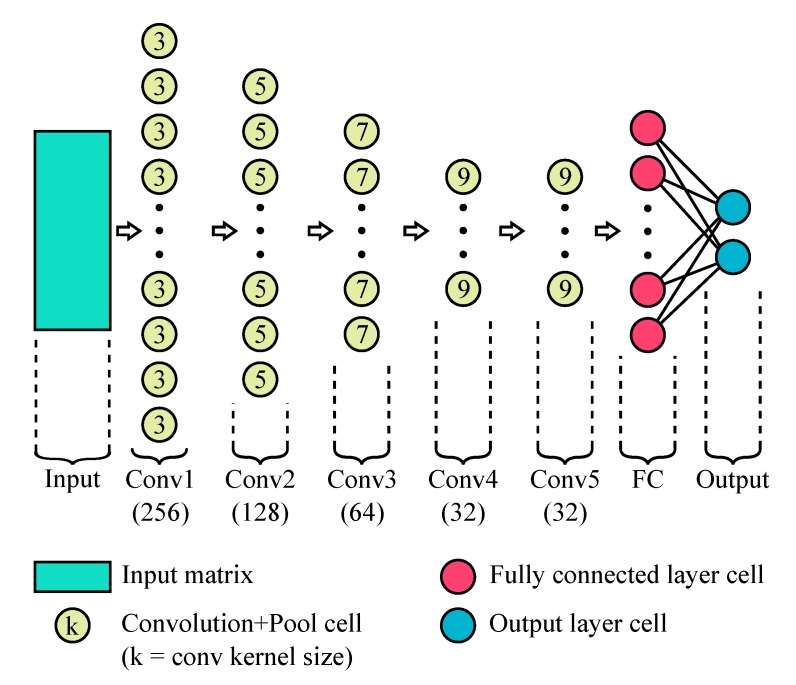
Description of the implemented CNN structure. The number between parenthesis below each convolutional layer represents the number of convolutional filters in that layer.

Based on our previous work [42], the chosen CNN structure consists of five convolutional layers, having 256, 128, 64, 32, and 32 filters with kernel sizes of 3, 5, 7, 9, and 9, respectively. After each convolutional layer, the ReLU activation function is applied followed by an average pooling layer with kernel size 2. The network ends with a fully connected layer followed by the output layer (Figure 5).

In order to allow our CNN to assess the MF state of operators, we need first to teach it how to recognize MF based on physiological signals. This process is called training, and it relies on assigning labels to the input data by relating the physiological data from sensor to different MF levels. We opted for using the Karolisnka Sleepiness Scale (KSS) [14] questionnaire to support the establishment of an MF scale. The KSS scale measures nine degrees of sleepiness, ranging from level 1 (very alert) to 9 (very sleepy, great effort to stay awake, fighting sleep). The KSS has being extensively used in fatigue and sleepiness-related studies. It has also been validated against physiological measures, including EEG and EOG [43].

**Figure 6:**
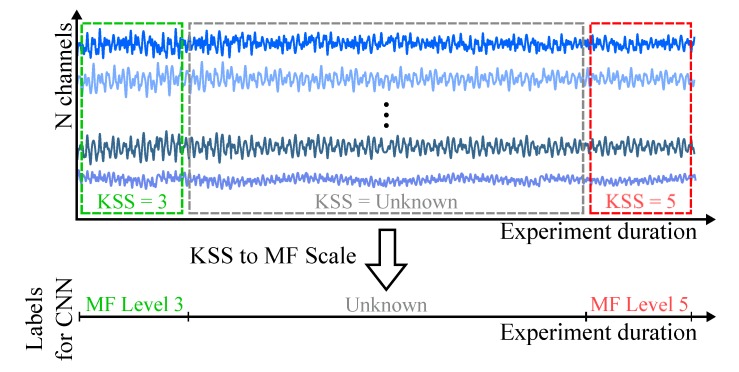
Labeling approach used to convert Karolisnka Sleepiness Scale (KSS) score to our proposed mental fatigue (MF) scale for CNN training.

We propose in this work the use of the KSS questionnaire to derive an MF scale for each participant. The MF scale is composed by two boundary MF levels and a variable number of transition states. The lowest KSS score obtained from one participant is used to define the first boundary level for that participant, the low MF condition. The highest KSS score is used to define the second boundary level, the high MF condition. The number of transition states depends on the number of KSS levels between the low and high MF levels. Figure 6 exemplifies how the data labeling is performed based on the KSS scores assigned by one participant. The data with unknown labels are not used for training the CNN, only to assess its generalization capabilities.

### 2.6. Experimental Setup

In order to evaluate the proposed framework, we performed a small-scale experiment. The experiment used a mixed method approach, where a scenario-based experiment was combined with a questionnaire.

**Figure 7:**
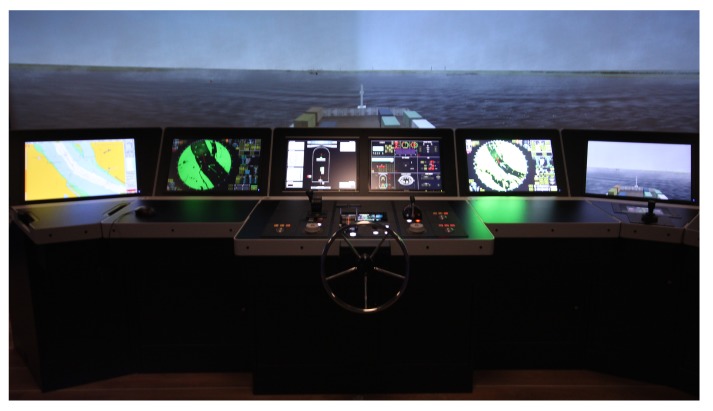
Experimental setup on vessel simulator, at the Norsk Maritimt Kompetansesenter.

The scenario-based experiment consisted of a simulated operation, designed to fatigue the participants. The simulated operation was carried out in vessel simulators used for training purposes. The simulator facilities are an accurate reproduction of a real vessel bridge, including all the commonly present controls, panels, and displays. Figure 7 shows the described simulator setup. The simulated task was monitored using the sensor framework described in Section 2.1. The simulated task consisted of navigating a vessel in a narrow canal area with heavy maritime traffic. The navigation was conducted at high speed (22 knots), and the pilots were supposed to overtake other vessels while properly navigating through the canal. Due to these requirements, the navigation demanded a lot of attention and care from the pilots. We designed the scenario to last between 60 and 90 min. The length of each run varied based on the approach each individual participant took to the task. We are aware that this task by itself is not representative of a complete shift of a pilot on board a vessel, which could last between 8 and 12 h. Our goal with the designed experiment was to reproduce a small portion of a pilot’s duties by using as case study a specific navigation task and show that the sensor framework could be used to detect the MF development even in a situation where extreme tiredness is not the case.

During the experiments, we monitored eleven volunteers, all male, aged 20 to 34 (23.91±3.89) years old. Among the volunteers, we had both trained and in-training pilots. The minimum requirements for the participants were knowledge about navigation rules, and how to pilot a vessel with the bridge layout present in the simulators. The experimental procedure was presented to the participants before the experiment and their informed consent was obtained. Ensuring the same baseline for all participants is challenging due to how differently MF develops and manifests in each person. In order to ensure a good baseline condition in the beginning of the experiments across all test subjects, participants also received orientation about sleep and the consumption of stimulants and alcohol prior to the experiment. Our experiment followed the principles and guidelines of the Declaration of Helsinki and participants’ data were handled following the recommendations of the Norwegian Centre for Research Data [44]. Part of the experiments were performed in the new vessel simulator facilities located at the Norsk Maritimt Kompetansesenter, in Ålesund, Norway. Another batch of experiments was performed in the simulator facilities at the Numerical Offshore Tank, in São Paulo, Brazil.

The KSS questionnaire was used by the participants to self-assess their tiredness state. During the process of collecting data to train the neural network, the operators assigned their tiredness state according to the KSS questionnaire before starting and after finishing the operation. This process was used to label the data, allowing the neural network to recognize which set of features in the physiological signals are related to each MF level. This labeling process is described in Section 2.5.

## 3. Results

The CNN training was done using a nested 20-fold cross validation approach. In each fold, 60% of the data were used as the training set, 20% as the validation set, and 20% as the test set. This approach is less biased if compared to one where the maximum accuracy obtained is taken as a representation of the network classification and generalization capabilities. In each fold, the training procedure was carried out for at least 15 epochs. The validation accuracy was the metric for adjusting the dynamic learning rate and termination criteria for the training. If after five epochs of training no improvement on the validation accuracy was obtained, the learning rate would be reduced by 20% and after 15 epochs without improvement the training process would be terminated. After termination, the network configuration with the best validation performance was used to evaluate the network test accuracy.

**Figure 8:**
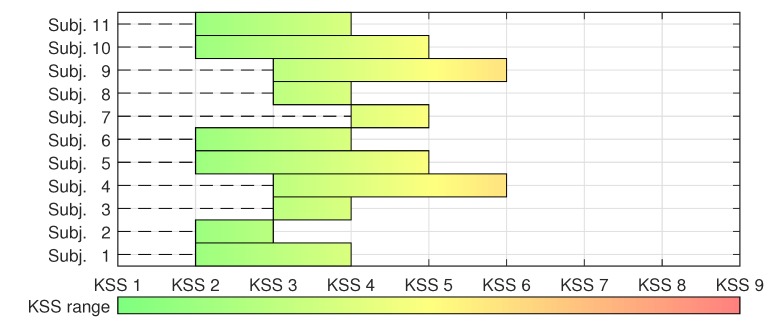
Reported KSS range of each test subject. For each subject, the lower limit shows the reported KSS level at the beginning of the experiment and the upper limit shows the reported KSS level at the end of the experiment.

In our experiment we monitored eleven test subjects and performed only single-subject analysis. For most subjects, we recorded data from ECG, temperature, EMG, and EEG sensors. For four subject we also recorded eye tracker data. These data were used as input for training the CNN to distinguish between low and high MF states. The data were labeled based on the answers for the KSS questionnaire at the beginning and end of the experiments. The results from the KSS questionnaire for all participants are presented in Figure 8. The figure shows in which range each participant assigned their own MF state during the experiments. This visual representation of the KSS range is used later on the paper in Section 4, when we present discussions about the generalization capability of the proposed CNN.

Some of the test subjects only reported a change of one unit in the KSS questionnaire response. Although this indicates a small progression in the MF level during the experiment, there data are still relevant in order to evaluate whether the proposed sensor framework is capable of differentiating MF states that are close to each other in the scale. This is especially relevant for ensuring that the system is capable of detecting the progressive transitions between different MF levels, as we are going to discuss in Section 4.

For each experiment we consider four different sensor configurations for the MF classification. Case 1 considers ECG, EMG, and temperature sensors. Case 2 uses as input only eye tracker data; namely, data from a three degrees of freedom accelerometer, data from a three degrees of freedom gyroscope, and right and left pupil diameter data. Case 3 comprises EEG data; specifically, the channels AF3, F3, O1, O2, F4, and AF4. Case 4 includes all available sensors for the CNN training. For Subjects 1 and 2, the EEG data were compromised and could not be used in the analysis. For Subjects 5–11, eye tracker data were not recorded due to the eye tracker sensor being unavailable at the time of the experiments. This situation is not ideal, but we still wanted to present some preliminary discussion about the use of the eye tracker sensor for this kind of MF analysis. Taking that into consideration, the cross comparison between cases which includes Case 2 should be considered with reservations. The test accuracies obtained by the trained CNN for each test subject and each case are presented in Table 1.

Analyzing the results for Subject 1, we can notice that Case 1 performed poorly in distinguishing between the low and high MF states, reaching 63% test accuracy. This indicates that the ECG, EMG, and temperature sensors by themselves were not sufficient for a good classification of MF state. Case 2 presents a better classification performance (82% test accuracy), which was relevant for achieving an also better classification performance on Case 4 (82% test accuracy). From the results of Subject 2, we can see that Case 1 presents a very good classification performance with 94% test accuracy, while Case 2 presents a good but worse performance, at 80% test accuracy. Once again we can see how the higher accuracy for one of the first two cases can help provide good classification performance in Case 4. This shows the corroborative power of sensor fusion, where one set of sensors can aid evaluation, even when another set provides incomplete or imprecise information.

Analyzing the results from Subjects 3 and 4, we observe similar results in both experiments. Cases 1 and 2 provide good classification accuracies, with Case 1 providing better accuracy than Case 2 for Subject 3 and with Case 2 providing better accuracy than Case 1 for Subject 4. For both Subjects 3 and 4, Case 3 has an almost perfect MF classification performance, reaching 99% test accuracy. When analyzing Case 4 for Subject 3 and Subject 4, we can see that the use of all available data actually worsened the classification performance. This demonstrates a competitive behavior of the sensor fusion system, where the use of discordant sensor information can degrade the performance of the assessment algorithm.

Looking at results from Subjects 5–11, we can see that Case 3 generally presented better classification accuracy than Case 1. It is also noticeable that in general, for each subject, Case 4 presents lower classification accuracy than the best classification accuracy between Cases 1 and 3, but it never performed worse than the worst case between Cases 1 and 3. In these cases, the fusion of different sensors data ensures that the worst performing case will be supported by the best performing one, providing a middle ground between the two extremes. This is especially relevant when we are not sure which case will present the best classification performance.

## 4. Discussion

### 4.1. Impact of Sensor Combination on CNN Generalization

The use of more diverse sources of information can lead to an increase in the generalization capability of the CNN by reducing its overfit to the training data. The generalization capability of a neural network refers to how well it performs when dealing with data to which it has never been presented before. In our case studies, we use the first and the last 20% of the data for the training process. The remaining 60% is used to evaluate the CNN generalization.

In our proposed method, a good generalization capability is not only linked to correctly assessing the non-fatigue and fatigue states. We are also interested in the transition between these two states. During the training procedure, we only used two different labels to train the CNN. In order to capture the transition phase, which is in essence additional labels, we apply a small trick. The trained CNN only performs consistently on the kind of data it was trained in. When presented data from an unknown class, the classification performance is inconsistent. This inconsistency can be transformed in the transition class by averaging the assessment results using a sliding window that covers the last 30 s of assessment. This process, used to produce the MF scale, is described in Algorithm 1.
**Algorithm 1** Mental fatigue assessment.1: **procedure** MENTAL FATIGUE SCALE2:     Continuously read and preprocess data from physiological sensors3: *loop every 4 s:*4:     Segment time-series with sliding window (length = 6 s/overlap = 2 s)5:     Fuse time-series segments into 2D input6:     Feed 2D input to CNN and get classification output7:     Obtain MF level by averaging CNN output with averaging window  (length = 5 steps)8:     Plot MF level

**Figure 9:**
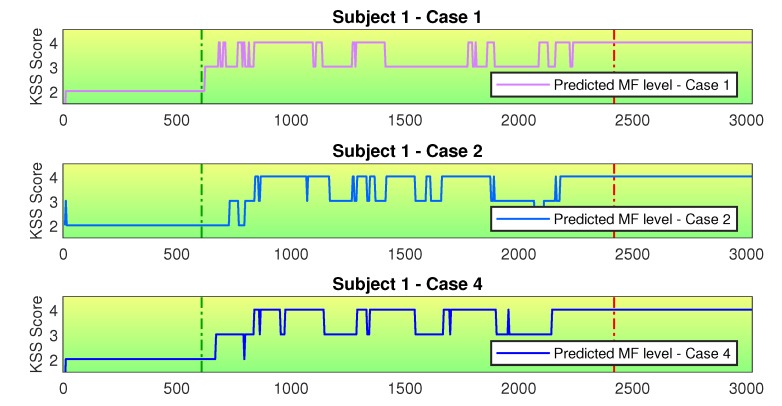
MF assessment for different cases for Subject 1. The horizontal axis is presented in seconds. The vertical green dashed line marks the superior limit of the data used for training the non-fatigue condition. The vertical red dashed line marks the inferior limit of the data used for training the fatigue condition. Different cases present good agreement among themselves.

In order to evaluate the generalization capabilities of the different sensor configurations we are going to discuss in more detail the assessment results of selected subjects. Figure 9 shows the different MF assessments for Subject 1. There is good agreement among the different cases, which shows that the generalization capabilities of the trained CNN is quite similar for all cases. We don’t expect to see an exact match between the different assessments, but we are interested in capturing the MF progression and the transition between the non-fatigue and fatigue states. In this case, there is essentially no difference between the assessment capabilities of the different sensor configurations.

**Figure 10:**
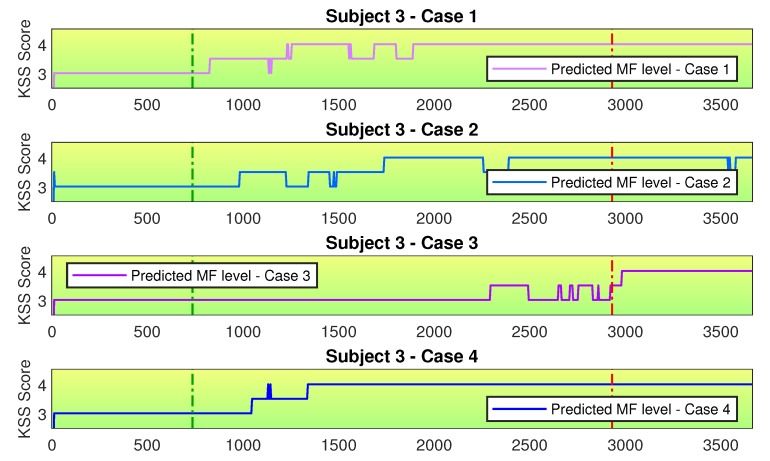
MF assessment for different cases for Subject 3. The horizontal axis is presented in seconds. The vertical green dashed line marks the superior limit of the data used for training the non-fatigue condition. The vertical red dashed line marks the inferior limit of the data used for training the fatigue condition. Case 3 produced a sharp transition between the fatigue and non-fatigue states. The addition of other sensors data helps with producing a smoother transition phase.

Figure 10 presents the different MF assessments for Subject 3. There is good agreement between Cases 1 and 2, but Case 3 presents a different transition phase. This difference happens due to the fact that the MF labels obtained from the KSS questionnaires can be imprecise. The EEG data carries a lot of relevant features for MF assessment, and training the CNN using this data makes the model learn a somewhat stiffer representation of the MF development, since even the imprecise labels can be learned as correct ones. In this case, transition features were learned as non-fatigue ones. As a consequence, the transition from non-fatigue to fatigue is modeled in a more abrupt way. This fast transition is not a good representation of the MF state progress, since the MF development process is a continuous phenomena, with gradual progress over time. When including other sensors data together with EEG data, Case 4 presents a smoother transition when compared to Case 3. As a result, the high fidelity to the training data obtained when using EEG data can be balanced by a better generalization capability when combining the EEG with other sensors.

**Figure 11:**
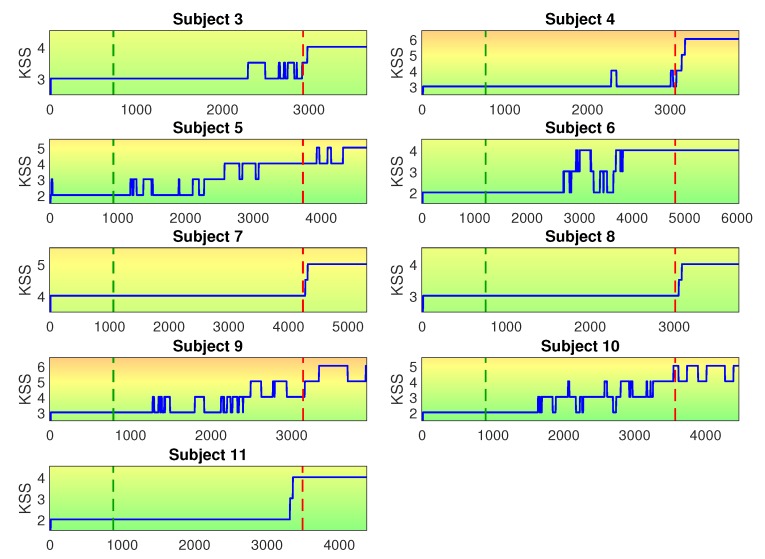
MF assessment for Case 3, for all available subjects. The horizontal axis is presented in seconds. The vertical green dashed line marks the superior limit of the data used for training the non-fatigue condition. The vertical red dashed line marks the inferior limit of the data used for training the fatigue condition. The EEG sensor presented the best classification performance, but transitions between different MF levels can be abrupt.

When considering which sensor configuration is the best, we need to consider the trade-off between framework complexity and assessment precision. Regarding framework complexity, the use of several physiological sensors can disturb the proper execution of the task being monitored. If this is the case, using only the best performing sensor (Case 3) would be the best option. Regarding assessment precision, there are two aspects to consider. First, the precision for distinguishing between the non-fatigue and fatigue states. In this case, the EEG sensor (Case 3) offers superior performance. Second, the characterization of the transition state. In this case, since the use of extra sensor data together with EEG data improves the representation of the transition between the non-fatigue and fatigue states, Case 4 would be the best configuration for performing the MF assessment task. It is important to remember that, for this case, we would not be interested on the absolute value in the MF scale in a specific instant for one subject. Instead, we are interested in the trend the overall assessment indicates. After all these considerations we conclude that the use of only the EEG sensor (Case 3) is, overall, the best option, since it combines high classification accuracy with a simplistic sensor setup. Figure 11 presents the MF assessment for Case 3, for all available subjects.

### 4.2. Looking Inside the CNN

One of the main issues related to the application of neural networks to decision making is the difficulty in understanding the reasoning behind the neural networks decisions. Due to its complex internal structure full of activation units and connections, deep neural network are commonly referred to as “black boxes”, since it is not trivial to visualize what kind of features from the input data the neural network is learning to recognize. In this work we are interested in understanding how our CNN model makes use of the physiological sensor data to perform the MF assessment task.

In order to understand which kind of feature is learned by each kernel of the network, we can apply a visualization approach based on deconvolution, proposed by [45]. This approach consists on projecting the activation of an specific kernel from an specific layer back to the input space. This is done by reversing the pooling and convolution operations performed by the CNN in each layer. The project activation can be then interpreted in the same domain as the original input. For the mathematical details of the implementation of this visualization approach, please refer to the original reference.

**Figure 12:**
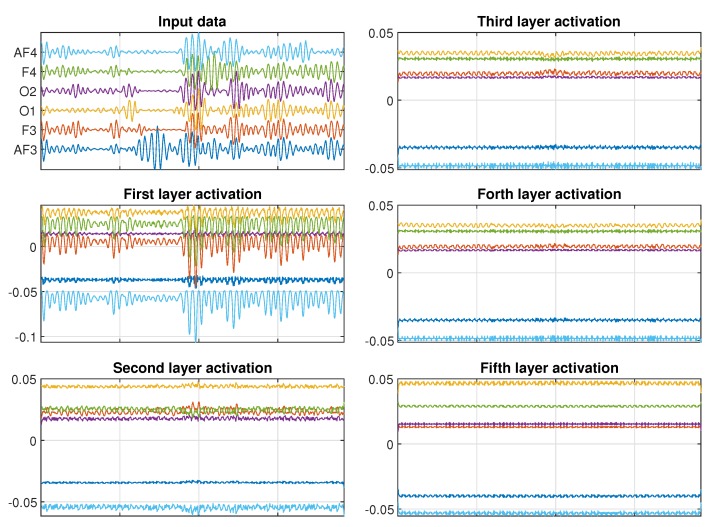
Projection of biggest activation of each CNN layer in the input domain for Subject 11 and KSS = 2.

As a case study, we are going to look at the CNN used to classify the MF state based on the EEG data for Subject 11. We can investigate which features the kernels with the biggest activation in each layer of the CNN model are important. Figure 12 shows a typical input data for the low MF case. The input data are composed by six EEG channels, sampled at 128 Hz and length of 6 s each. Figure 12 also shows the projection of the highest activations on each layer back to the input domain. We can see that for the first layers, the CNN learn features related to the amplitude of oscillation of each channel. As we look into deeper layers, the features cease to be related to amplitude start to be related to frequency. As an example, in the fifth layer, there is no signal of the input amplitude in the activations, but we notice that there is an oscillation between 12 and 16 Hz, which correspond to part of the Alpha and Beta frequency band of the EEG signal. We can also see that, for this specific subject, the CNN considers the channels F4 and O1 more relevant for distinguishing between the different MF states, since they appear with higher activation values when projected to the input domain.

**Figure 13:**
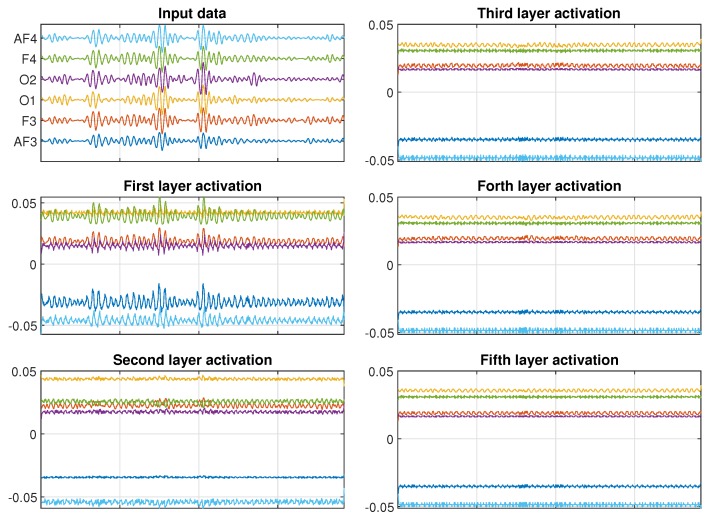
Projection of biggest activation of each CNN layer in the input domain for Subject 11 and KSS = 4.

Figure 13 shows a typical input data for the high MF case. Similarly to what we observed from the low MF case, the first CNN layers present amplitude related features. Once again, the amplitude features fade away in the deeper layers, which are dominated by the frequency related features. As for the low MF case, the CNN considers the channels F4 and O1 more relevant for distinguishing between the different MF states. When comparing Figure 12 and Figure 13, it is noticeable that the intermediate layers present very similar activations. But when comparing the fifth layers, we can notice a significant difference in the activation of channel O1, which is 20% smaller for the high MF case. So, we can conclude that, for this specific participant, changes in the EEG channel O1 are the most relevant aspect for distinguishing between the different MF states. Due to the physiological difference between people, this result can’t be directly extrapolated to all other subjects, since variations on the activation of different channels can occur.

### 4.3. Limitations

As acknowledged throughout the paper, the presented study has some limitations. First, although we focused on MF detection in the maritime domain, we only performed experiments in a simulator environment. Although this limits the realism of the studied scenario, it also give us the security to experiment with different framework configurations without any associated risk. Second, we have problems related to the completeness of data available for the study. That problem is the most evident for the eye tracker data, since the sensor was only available for collecting data for four experiments. This weakens the cross-comparison between the different cases. Third, the limited number of participants and the reduced number of MF states reported by them are a relevant limitation to this study, since it limits our capacity to develop a broader and more precise MF scale. We aim to perform a larger scale test with longer session to increase the range of MF states reported by the operators. Additionally, self-assessment of MF can be performed during the operation. The addition of such intermediate MF states would help to create a more continuous classification algorithm. Fourth, the use of self-assessment of MF can lead to biased data labeling, and makes it difficult to establish a strong baseline across different test subjects. More objective methods such as the direct calculation of features from the physiological data can be combined with the self-assessment questionnaires to better support the data labeling process.

## 5. Conclusions

Assessing MF in real time during demanding maritime operations is an important and relevant human-factor challenge in the maritime domain. Maritime accidents affect all the main stakeholders involved in such operations, most importantly the people working on board, but the environment and the maritime industry as a whole too. The lack of a trustworthy method with which to assess MF in maritime operators in order to avoid accidents due to human error motivated this research. In order to tackle this issue, we proposed the use of a physiological sensors framework for assessing MF in demanding maritime operations. The MF assessment is performed by a CNN and the data labeling is achieved with the use of the KSS questionnaire.

The framework was implemented and tested in a vessel simulator. This experiment was performed as a proof of concept where we wanted to explore different sensor configurations and demonstrate the framework’s feasibility and applicability as an MF assessment tool. During our experiments we managed to observe that, although all tested sensor configurations are capable of detecting different MF levels, some configurations are preferable, depending on the main focus of the assessment framework. If intrusiveness is a concern, a configuration with fewer sensors is preferable. In this case, the EEG or eye tracker are the best options. For a high classification precision based on the labeled data, the EEG presented the highest test accuracy. For capturing smoother transitions between different MF levels, the configuration using all sensors performed better on assessing the transition states. In the end we are left with several feasible sensor configurations, and a sensor framework which performs as intended and can be tailored to adapt to specific MF assessment needs.

Once trained, the CNN algorithm can be applied in real time, with little computational effort. The algorithm can capture not only the non-fatigue and fatigue states, but also the gradual MF build up process. A natural next step for this research is the investigation of real-world scenarios in order to assess the trade-off between the framework’s intrusiveness and the benefit it brings to the safety of maritime operations. For performing this analysis, the system needs to be expanded, and a risk assessment methodology based on the operators’ MF levels need to be developed.

## Figures and Tables

**Table 1:** MF classification results using a CNN with different sensor configurations. The different case configurations are as follows: Case 1—EMG, temperature, ECG; Case 2—eye tracker; Case 3—EEG; Case 4—all available sensors.

	Case 1	Case 2	Case 3	Case 4
	Test acc (avg ± std)	Test acc (avg ± std)	Test acc (avg ± std)	Test acc (avg ± std)
Subj. 1	0.63 ± 0.13	0.82 ± 0.03	-	0.82 ± 0.04
Subj. 2	0.94 ± 0.02	0.80 ± 0.05	-	0.93 ± 0.04
Subj. 3	0.93 ± 0.03	0.77 ± 0.04	0.99 ± 0.01	0.95 ± 0.02
Subj. 4	0.81 ± 0.05	0.88 ± 0.04	0.99 ± 0.01	0.90 ± 0.05
Subj. 5	0.94 ± 0.03	-	0.95 ± 0.03	0.96 ± 0.02
Subj. 6	0.99 ± 0.01	-	0.98 ± 0.02	0.99 ± 0.01
Subj. 7	0.97 ± 0.02	-	1.00 ± 0.01	0.97 ± 0.02
Subj. 8	0.84 ± 0.07	-	0.99 ± 0.02	0.85 ± 0.08
Subj. 9	0.66 ± 0.13	-	0.91 ± 0.04	0.87 ± 0.04
Subj. 10	0.94 ± 0.03	-	0.98 ± 0.02	0.96 ± 0.02
Subj. 11	0.81 ± 0.18	-	1.00 ± 0.00	0.81 ± 0.17

## Abbreviations

The following abbreviations are used in this manuscript:

CNN	convolutional neural network
ECG	electrocardiogram
EEG	electroencephalogram
EMG	electromyogram
EOG	electrooculogram
KSS	Karolisnka Sleepiness Scale
MF	mental fatigue
